# Force-Field Benchmark for Polydimethylsiloxane: Density, Heat Capacity, Isothermal Compressibility, Viscosity and Thermal Conductivity

**DOI:** 10.1021/acs.jpcb.4c08471

**Published:** 2025-02-03

**Authors:** Zhirui Xiang, Chao Gao, Teng Long, Lifeng Ding, Tianhang Zhou, Zhenghao Wu

**Affiliations:** † Department of Chemistry and Materials Science, Xi’an Jiaotong-Liverpool University, Suzhou 215123, Jiangsu, P. R. China; ‡ Aerospace Research Institute of Materials & Processing Technology, Beijing 100076, P. R. China; ¶ School of Materials Science & Engineering, Shandong University, Jingshi Road, 17923 Jinan, Shandong, China; § College of Carbon Neutrality Future Technology, State Key Laboratory of Heavy Oil Processing, China University of Petroleum (Beijing), Beijing 102249, P. R. China

## Abstract

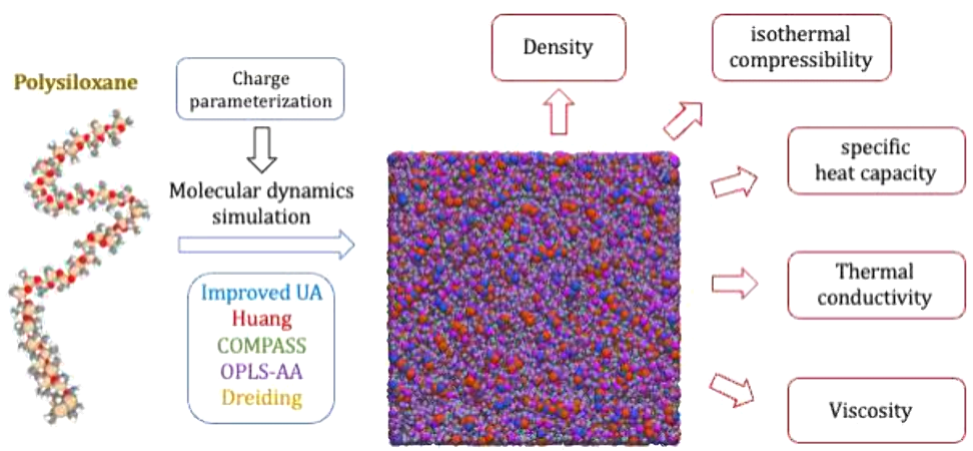

Polysiloxanes are versatile polymeric materials with widespread applications in industries ranging from electronics to biomedical devices because of their unique thermal and viscoelastic properties. Accurate molecular simulations of polysiloxanes are essential for understanding their broad applications from a microscopic perspective. However, the accuracy of these simulations is highly dependent on the quality of the force fields used. In this work, we present a comprehensive benchmark and development of force fields tailored for polydimethylsiloxane, which is one of the most widely used polysiloxane materials. Our focus is on their performance in predicting key thermophysical properties including density, heat capacities, isothermal compressibility, and transport properties such as viscosity and thermal conductivity. Experimental measurements are performed in parallel to rigorously validate simulation outcomes. Existing force fields for polydimethylsiloxane, including those derived for organic and inorganic systems, are systematically evaluated against experimental data to identify limitations in accuracy and transferability. Simulation results are compared extensively with experimental observations across a range of temperatures and pressures, revealing the strengths and shortcomings of these commonly utilized force fields for polydimethylsiloxane. Discrepancies between force field predictions and experimental measurements are analyzed in detail for thermodynamic and transport properties of polydimethylsiloxane. This benchmark study serves as a critical assessment of current force fields for polydimethylsiloxane and offers guidelines for their further development, enabling more reliable simulations of polysiloxane-based materials for various industrial applications.

## Introduction

Polysiloxanes, a class of polymers with the general formula [R2SiO]n, where R can be a variety of organic groups, are renowned for their unique properties of thermal stability, low glass transition temperatures, and biocompatibility. These properties render them indispensable in a wide range of industrial and medical applications, including sealants, adhesives, and medical implants.^1−6^ To enhance the performance of polysiloxane-based materials and to tailor their properties for specific applications, a detailed understanding of their physical properties is essential. Traditionally, these properties are determined experimentally, which can be time-consuming and costly. Computational methods, particularly molecular simulations, offer an attractive alternative, providing a means of predicting properties under a wide range of conditions with relative ease and precision.^7−12^ Additionally, molecular simulation allows for microscopic understanding of the molecular mechanisms in complex behaviors of polymers, which is important for the development of novel functional polysiloxane-based materials.

The quality of molecular simulation predictions is based on the quality of the force field, a set of mathematical equations that describe the interactions between atoms within a molecule and between different molecules.^13^ A well-parametrized force field can ideally predict the macroscopic properties of a material from its microscopic structure through molecular simulations. In the context of polydimethylsiloxane (PDMS), several force fields have been developed,^7,13−16^ each with its own merits and limitations. One of the first attempts to model PDMS using MD simulations was made by Sok et al.^7^ who developed a Class I force field for PDMS systems to study the transport of small molecules across a PDMS membrane. Using relatively simple functional forms for the force field, their simulation predictions yielded good agreement of the diffusion coefficient compared to experimental data, while significant discrepancies in the solubility coefficient.

Sun developed a consistent atomic force field (referred to as COMPASS) that aims to be applicable to a wide range of chemistries.^13^ This Class II force field is built on the basis of quantum chemistry calculations of molecular systems, and its parameters are systematically scaled to fit the experimental data such as density and heat capacity. COMPASS was used for the prediction of the thermodynamic, structural, and vibrational properties of PDMS.^17^ Reasonable agreement with the experiment was observed in all cases. Curro and co-workers developed a refined force field at the unit-atom level to extend the accessible spatiotemporal scales of MD simulations for PDMS materials.^15^ Good agreement was reported between experiments and simulations in pressure and structural properties.^15^ Later, Smith et al. developed a modified version of the Class II force field based purely on quantum chemistry using more complex oligomers of PDMS and at a higher level of quantum chemistry calculations.^18^ Recently, Huang et al. developed an improved Class II force field for PDMS systems by combining bottom-up and top-down approaches using both quantum mechanical calculations and experimental data.^14^ Huang’s model demonstrated good predictions on thermodynamic properties such as density, solubility parameter, and glass transition temperatures.

Although these force fields have shown effectiveness in predicting certain material properties, many remain insufficiently validated for other thermodynamic and transport characteristics. It is crucial to assess the precision and reliability of these force fields in predicting the properties of common materials within MD simulations.^19,20^ However, a comprehensive benchmarking study for PDMS systems has yet to be conducted. Moreover, a critical issue remains as to whether these models that are parametrized with certain experimental data relevant to thermodynamics can reliably predict other key properties in dynamics and rheology, such as viscosity and thermal conductivity. Nevertheless, the challenge of transferability continues to pose difficulties in the force field development community. Extensive and systematic benchmarks are genuinely required for conducting reliable and accurate molecular simulations of PDMS materials.

In this work, we performed comprehensive benchmarks of PDMS force fields to evaluate their performance in predicting various thermodynamic and transport properties through molecular simulations. Considering their diverse characteristics and parametrization levels, five force fields were employed, namely, united-atom model from Frischknecht and Curro,^15^ improved Class II model from Huang et al.,^14^ COMPASS,^13^ OPLS-AA,^19^ and Dreiding.^21^ Among these models, the Frischknecht’s united-atom model and Huang’s all-atom model are both specifically parametrized for PDMS. Three universal force fields, i.e., COMPASS, OPLS/AA and Dreiding, are also included for comparison. Despite their widespread use, these force fields differ significantly in their parametrization processes, particularly in their relevance to polydimethylsiloxane (PDMS) systems. The COMPASS force field^13^ incorporates specific parametrizations tailored for siloxane chemical environments, enabling it to accurately capture a broad spectrum of molecular interactions. In contrast, the Dreiding^21^ and OPLS-AA^19^ force fields, although general-purpose all-atom models designed for organic molecules, lack explicit descriptions of siloxane-specific interactions. Consequently, we recalculated the partial charges for these models to enhance their applicability to PDMS systems, ensuring a more accurate representation of the unique chemical environment. We systematically investigate a broad spectrum of thermodynamic and transport properties of PDMS systems, including density, specific heat capacity, isothermal compressibility, viscosity, and thermal conductivity, to assess the precision, reliability, and applicability of five distinct force fields. Through extensive MD simulations, we evaluate the predictive accuracy of these force fields under varying temperature and pressure conditions. The benchmarking process involves a detailed comparison of simulation results with experimental data, enabling the identification of each force field’s strengths and limitations. Furthermore, we examine the sensitivity of the predicted properties to variations in force field parameters, providing valuable guidance for the refinement and development of force fields specifically optimized for polysiloxanes.

## Computational Methods

### PDMS Force Fields and Simulation Details

In this study, we systematically examine five PDMS models at both all-atom (AA) and united-atom (UA) resolutions, assessing their performance across a wide range of thermodynamic and transport properties. These force fields fall into two categories based on their development approach: (1) general-purpose universal force fields and (2) those specifically optimized for PDMS systems. Among the models evaluated, OPLS-AA, COMPASS and Dreiding represent the former category, while latter category includes models like the Huang’s model and the UA force field. Key features of these force fields have been summarized in Table 1. Detailed force-field parameters and relevant scripts for simulations can be found in reference.^22^ The force-field parameters for each model are employed according to their original development. Notably, the original OPLS-AA model lacks parameters for Si–O interactions. To address this issue, we perform additional partial charge optimization using two distinct methods: Charge equilibration (Qeq) method^23^ and density-functional-theory calculations.^24^ The partial charges for Si and O atoms derived from the Qeq method are selected due to their superior performance (as detailed in the Discussion section). Accordingly, all results presented in the Results section are based on this charge parametrization. The Moltemplate software^25^ is used to generate the topology and parameters for the universal force fields, i.e., OPLS-AA, COMPASS and Dreiding, while the Huang’s and UA models are created manually with parameters extracted from the original paper. In detail, a uniform cutoff distance of 1 nm is applied to COMPASS, UA, and dreiding (HUANG model uses 1.2 and OPLS uses 1.1) for nonbonded interactions. This same distance is also used as the switching distance in the Particle–Particle Particle-Mesh (PPPM) algorithm^26^ for calculating long-range Coulomb interactions in the all-atom models. More details about the force-field input parameters are available in the Supporting Information.

**Table 1:** Summary of Force Fields Used in This Work

Force Field	Type	Key Features
OPLS-AA	All Atom	Class I; universal force field; optimized for organic liquid simulations
Dreiding	All Atom	Class I; universal force field; includes H-bond interactions
COMPASS	All Atom	Class II; universal force field; includes siloxane environments in parametrizations
HUANG	All Atom	Class II; specifically parametrized for PDMS
UA	United Atom	Hybrid Class I and Class II; specifically parametrized for PDMS

All simulations are performed using the LAMMPS (Large-Scale Atomic/Molecular Massively Parallel Simulator) package with periodic boundary conditions applied in the *x*, *y*, and *z* directions.^27^ The initial configurations of the systems are created using the Packmol,^28^ where polymer chains are randomly placed in a large cubic box. The details of the simulated systems are given in Table 2. The chosen system size was evaluated and found to have a minor effect on the calculated properties, such as viscosity and thermal conductivity, as illustrated in Figure S3. The simulation workflow consists of three main stages: system generation, equilibration, and production. In the generation stage, the initial configuration is set at *T* = 300 K and *P* = 100 atm under the *NPT* ensemble. The high pressure in this step is used to force the chains into the liquid phase. The pressure is then gradually reduced to *P* = 1 atm, and the system is allowed to pre-equilibrate at this pressure until the density converged. This is followed by a 1 ns equilibration phase under the *NPT* ensemble at *T* = 300 K and *P* = 1 atm. Equilibration is monitored by observing the fluctuation of the total energy within a small variation. The time evolution of the total energy of two example systems during the equilibration stage can be found in Figure S1. After equilibration, production runs are conducted to gather data for property calculations. It is noted that, for computing viscosity, the system is considered equilibrated when the autocorrelation function of chain end-to-end vector decays to zero. Three distinct types of production run are employed, each designed to calculate particular properties, as detailed in Table 3 and the following sections. It is important to note that because of the varying relaxation times of the system at different temperatures, the durations of the production runs can differ. The Nóse–Hoover thermostat and barostat are employed with coupling constants of τ_*P*_ = 1 ps and τ_*T*_ = 0.1 ps, respectively. The equations of motion are integrated using the velocity-Verlet algorithm with a time step of *δt* = 1 fs for all models. Additional details on the simulation setup can be found in Supporting Information.

**Table 2:** System Information for Different Properties Calculation

Property	No. of Monomers	No. of Chains	Temperature (K)
Density, Specific Heat Capacity, Isothermal Compressibility	50	64	250–450
Thermal Conductivity	50	125	250–450
Viscosity	11	100	300–450

**Table 3:** Details of Different Types of Production Runs in the Simulations

Type	Target Properties	Key Features	Ensemble	Length
Type I	Density, Heat Capacity, Isothermal Compressibility	Equilibrium MD	*NPT*	10 ns
Type II	Thermal Conductivity	Nonequilibrium MD	*NVE*	1 ns
Type III	Viscosity	Equilibrium MD	*NVT*	10 ns

### Analysis of Thermodynamic and Transport Properties

#### Thermodynamic Properties

Subsequent to the equilibration, the Type I production is performed to compute the thermodynamic properties. Specifically, a 10 ns production run is conducted under the *NPT* ensemble, with the thermodynamic output, such as mass density, volume of simulation box, and potential energy, recorded every 1 ps. As one of the most common thermodynamic properties of PDMS materials, the mass density is calculated from simulations following the equation:
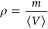
1where *m* and *V* represent the total mass and the volume of the simulation box, respectively; The bracket ⟨·⟩ denotes the ensemble average over the production run. In experiment, the density of PDMS (*M*_*w*_ ≈ 3700 g/mol with polydispersity index PDI ≈ 1.1) is measured using the vibrating tube method, with the equipment model of Anton Paar DMA 5000M. More details on experimental measurements performed in this study are given in the Supporting Information.

The isobaric specific heat capacity is another critical thermodynamic property that measures the amount of heat required of a substance by one degree (Kelvin) at constant pressure. From the 10 ns production run, we calculate the isobaric specific heat capacity *C*_*p*_ using the formula displayed below:
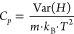
2where *m* is the total mass of the system, *k*_B_ is the Boltzmann constant, and *T* is the temperature in units of Kelvin (K). The symbol Var(*H*) represents the variance of the enthalpy. The enthalpy *H* is computed as *H* = *U* + *PV*, where *U* is the total potential energy of the system and *P* and *V* are the pressure and volume of the simulation system, respectively.

The isothermal compressibility κ_*T*_ is a measure of how easily a material can be compressed at a constant temperature, providing insights into the material’s mechanical response. Based on thermodynamics, the isothermal compressibility is defined as
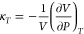
3where *V* is the volume of the system, *P* is the pressure, and the partial derivative (∂*V*/∂*P*)_*T*_ is taken at constant temperature *T*. In MD simulations, the isothermal compressibility κ_*T*_ can be calculated using the fluctuations in the volume and pressure of the system over time in the *NPT* ensemble. The relationship between volume fluctuations and isothermal compressibility is given by
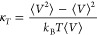
4Here, ⟨*V*^2^⟩ and ⟨*V*⟩^2^ are the mean-square volume and the square of the mean volume, respectively, *k*_B_ is the Boltzmann constant, and *T* is the absolute temperature. The same Type I production run of 10 ns is employed for the calculations.

#### Transport Properties

Transport properties of materials, such as heat transfer, i.e., thermal conductivity, and momentum transfer, i.e., viscosity, are of critical importance to understand material performance in applications such as energy efficiency, thermal management, and flow behavior in both industrial and technological processes. In this study, the thermal conductivity and viscosity are selected for benchmarking.

**Figure 1:**
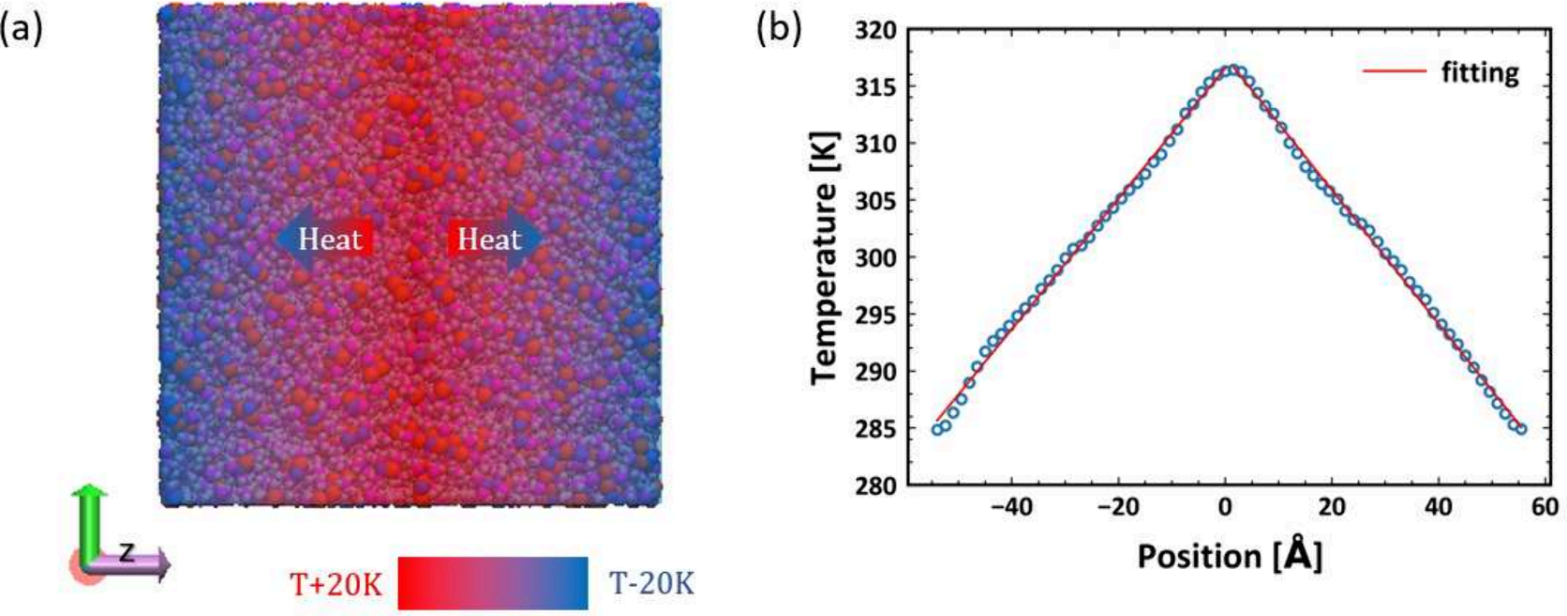
(a) System configuration for thermal conductivity calculation, rendered by VMD. (b) Example temperature profile averaged over 1 ns at steady state.

To calculate the thermal conductivity from MD simulations, we perform Type II production runs of nonequilibrium molecular dynamics (NEMD), following the equilibration stage. During the NEMD simulation, a constant energy per unit of time is introduced via a heat source (*T*_*h*_ = *T* + 20 *K*, 10 Å), positioned at the center of the simulation box along the *z*-axis. At the same time, energy is subtracted from two heat sinks (*T*_*l*_ = *T* – 20 *K*, each 5 Å in size) placed at the edges of the box, as shown in Figure 1 (a). The velocity rescaling method is used to maintain the desired temperatures at the heat source and sinks. A steady state with a stable temperature gradient is achieved after 10 ns NEMD. Subsequently, additional production runs are performed to calculate the thermal conductivity κ using the Fourier’s law:
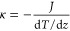
5where *J* is the heat flux at the steady state and d*T*/d*z* is the temperature gradient. An example of the system configuration and temperature profile in the steady state is shown in Figure 1. The NEMD simulations are performed at a constant volume (*NVT*) using the box dimension averaged over the last half period from the equilibration step. Three replicas of NEMD production runs starting from different equilibrium configurations are performed to compute the standard deviations that are taken as error bars. In experiments, the thermal conductivity of PDMS (*M*_*w*_ ≈ 3700 g/mol with PDI ≈ 1.1) is measured using the transient hot-wire method, with the equipment model DRL-III thermal conductivity tester from Xiangtan Xiangyi Instrument Co. Ltd. The transient hot-wire method involves using a thin wire as a heat source to apply heat to the material for a specific duration. As the wire heats up, it raises the temperature of the surrounding material. By monitoring the temperature change of the wire, the thermal conductivity of the material can be calculated.

We employ the Green–Kubo method^29^ to calculate the viscosity of PDMS materials from the equilibrium MD simulations of Type III production run. This approach utilizes the time correlation function of the off-diagonal elements of the stress tensor, which is related to the viscosity of the system. The viscosity η is then computed from the Green–Kubo relation given by
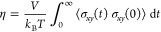
6where *V* is the volume of the simulation box, *k*_B_ is the Boltzmann constant, *T* is the temperature, and ⟨σ_*xy*_(*t*) σ_*xy*_(0)⟩ is the time correlation function of the off-diagonal components of the stress tensor σ_*xy*_. This method provides a rigorous way to extract the viscosity from MD simulations by capturing the response of the system to shear stress.^30,31^ The experimental measurement of the viscosity is performed using the rotational method, with the equipment model Haake rheometer Mars60, at a shear rate of 40 1/s. More details can be found in the Supporting Information.

## Results

### Thermodynamic Properties

**Figure 2:**
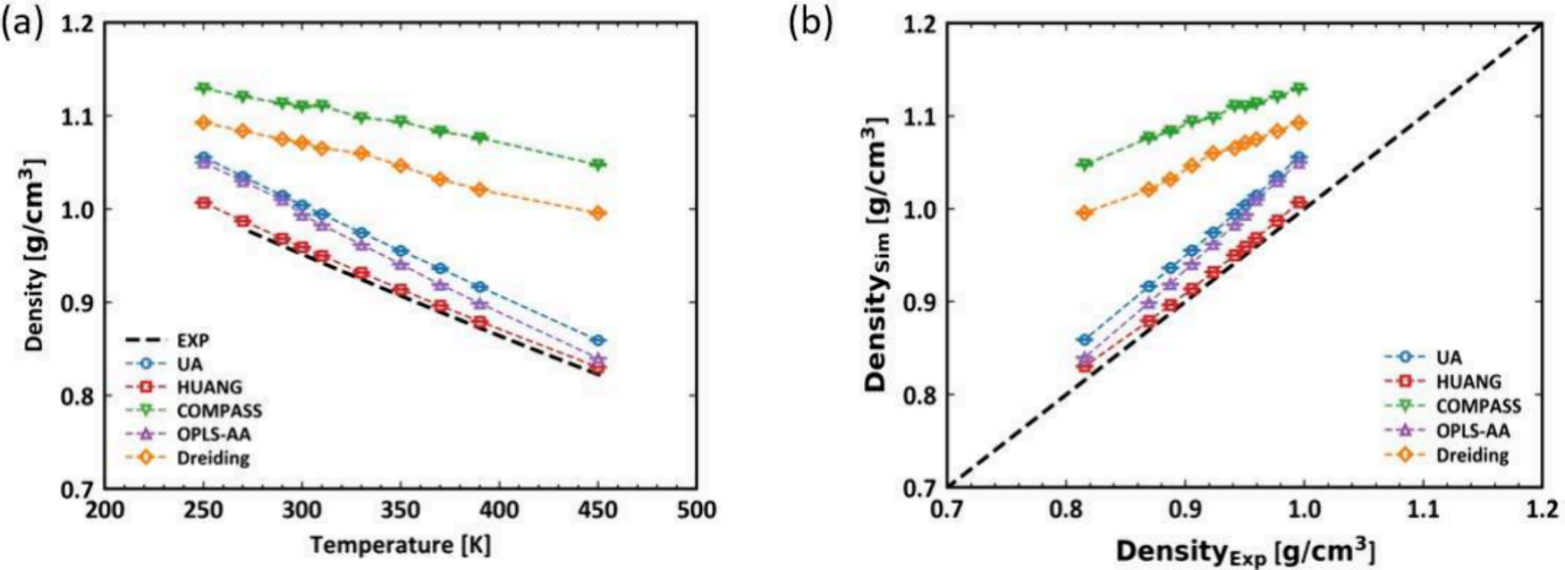
(a) Bulk density as a function of temperature of PDMS and (b) correlation between density calculated by MD simulations with UA (blue), Huang (red), COMPASS (green), OPLS-AA (purple) and Dreiding (orange) force fields under ambient pressure *P* = 1 atm. Corresponding experimental measurements (black dashed line) are also included for comparison.

Figure 2(a) illustrates the behavior of the simulated density of PDMS systems under different force field models compared to the experimental measurements. All five models examined in this work demonstrate a decrease in density with increasing temperature, consistent with the expected thermal expansion of the PDMS observed in the experiments. Specifically, the UA model significantly overestimates the density in the temperature range, displaying a larger deviation as the temperature increases. The Huang’s model follows the experimental measurements more closely but still shows slight overestimation at higher temperatures. The systematic overestimation observed at high temperatures in Huang’s models is likely attributed to the parametrization process, wherein the nonbonded parameters were optimized to reproduce experimental data at room temperature.^14^ The universal force fields generally behave worse than the former specific models, which aligns with the expectation. In detail, the COMPASS model consistently overestimates the density, maintaining the largest deviation from the experimental values among the force fields examined. The OPLS-AA model and the Dreiding model also overestimate the density, with OPLS-AA providing a closer match to the experimental data than Dreiding, especially at higher temperatures. Overall, the five models exhibit varying degrees of accuracy in reproducing experimental density values. The HUANG, UA, and OPLS-AA models demonstrate relatively good agreement with experimental data, whereas the Dreiding and COMPASS models display notable deviations.

**Figure 3:**
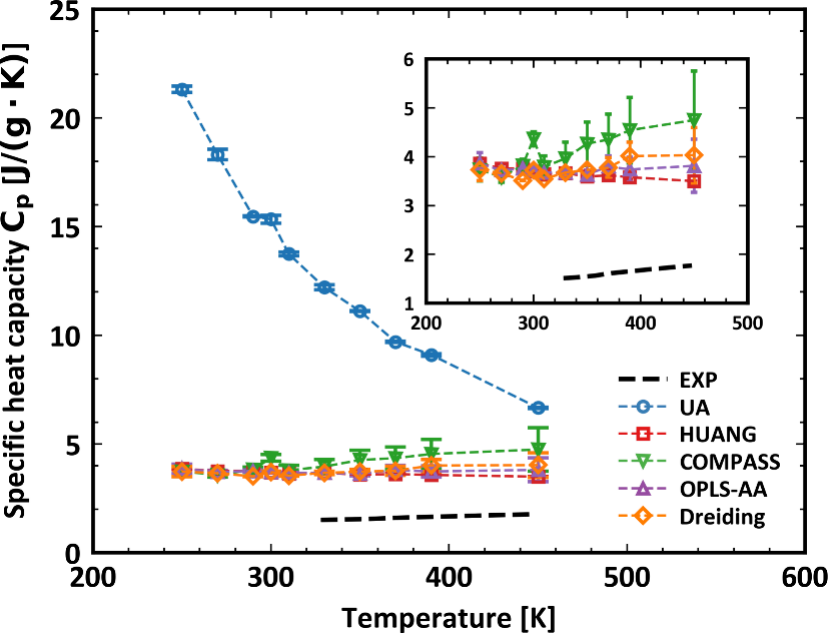
Specific heat capacity as a function of temperature of PDMS simulated.

The system setup for the specific heat capacity calculation is identical to that used for density, consisting of 64 PDMS chains, each containing 50 monomers. Figure 3 presents the temperature-dependent isobaric specific heat capacity (*C*_*p*_) for PDMS measured in J/(g ·K) over a temperature range of 250 to 450 K. Specifically, it displays the *C*_*p*_ trends derived from experimental data (EXP) and various simulation models, including UA, HUANG, COMPASS, OPLS-AA, and Dreiding force fields. The experimental data, depicted by the black dashed line, exhibits a gradual linear increase in *C*_*p*_ across the temperature range. In contrast, the UA model, represented by blue dashed lines with circular markers, shows a distinct and sharp decline in *C*_*p*_ as the temperature rises from 200 K to around 450 K. Other simulation models, i.e., HUANG, COMPASS, OPLS-AA, and Dreiding, show relatively constant *C*_*p*_ values with slight fluctuations across the temperature range. The inset figure zooms in on the lower *C*_*p*_ range (approximately 2 to 6 J/(g ·K)), highlighting the subtle variations among the HUANG, COMPASS, OPLS-AA, and Dreiding models, all of which remain above the experimental values. The data suggests significant deviations between the experimental measurements and the predictions by the UA model, as well as modest discrepancies among the remaining simulation models. This can be attributed to the reduced degrees of freedom in the UA model, where multiple atoms are represented by a single interaction site. This simplification affects the calculated vibrational modes and their contributions to the specific heat capacity. Consequently, the UA model overestimates the system’s thermal energy storage capacity, resulting in higher specific heat capacity values compared to those predicted by all-atom models. This comparison underscores the varying accuracy of different force field models in predicting thermodynamic properties such as specific heat capacity. It is noted that Caleman et al. reported a similar overestimation of the heat capacity of organic liquids when using the OPLS-AA force field, which was attributed to the neglect of nuclear quantum effects in the classical sampling approach employed in MD simulations.^20^

**Figure 4:**
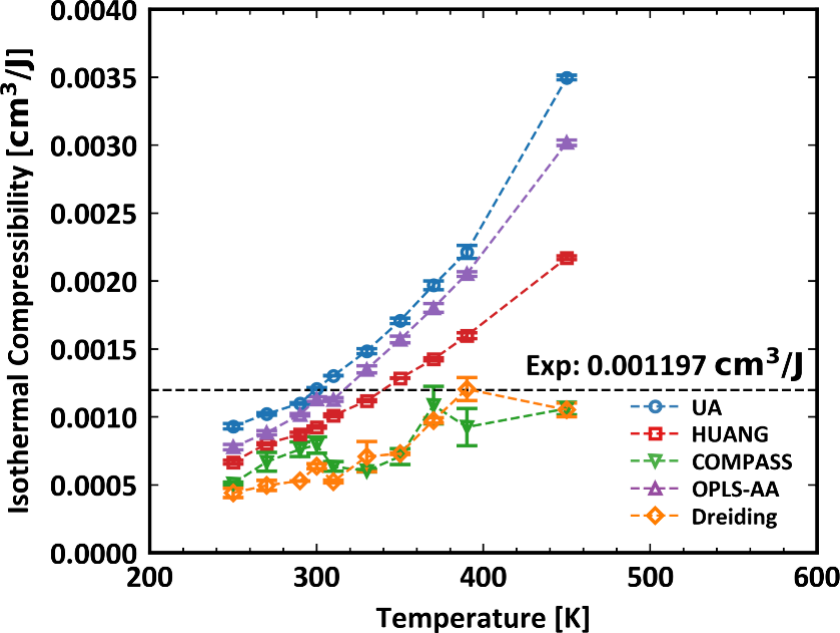
Isothermal compressibility as a function of temperature of PDMS simulated. The dashed line is the experimental data measured at 298.15 K.

The isothermal compressibility κ_*T*_ as a function of temperature for PDMS systems simulated with various force fields is shown in Figure 4. At low temperatures (200–300 K), all models underestimate κ_*T*_. As the temperature increases, the UA, HUANG and OPLS-AA models show a rapid increase in compressibility, with the UA force field showing the largest increase. Compared to the experimental data available that measure *T* = 298.15 K by Shih et al.,^32^ the UA model demonstrates the best predictions in κ_*T*_. Conversely, COMPASS and Dreiding models exhibit more moderate increases, staying below the experimental data measured at *T* = 298.15 K over the whole examined temperature range. These results clearly indicate the discrepancies between force field predictions and experimental values, highlighting once again the need for improved force-field accuracy of MD predictions in thermodynamic properties.

### Transport Properties

#### Thermal Conductivity

**Figure 5:**
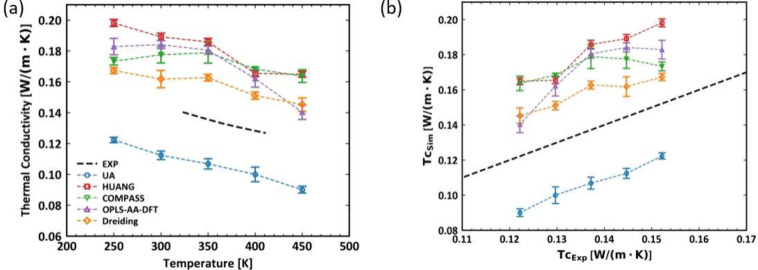
(a) Thermal conductivity as a function of temperature of PDMS simulated and experimental results and (b) correlation between thermal conductivity calculated from simulations with UA (blue), Huang (red), COMPASS (green), OPLS-AA (purple) and Dreiding (orange) force fields under ambient pressure *P* = 1 atm. Corresponding experimental measurements (black dashed line) are also included for comparison.

Figure 5 (a) displays the thermal conductivity of PDMS as a function of temperature from MD simulations with five different force field models: Dreiding, COMPASS, UA, OPLS-AA, and HUANG. The experimental measurements are also included for comparison. Two key observations can be made from this figure. First, the UA model is the only force field that consistently predicts lower thermal conductivity values than the experimental data throughout the entire temperature range examined in this study. Second, with the exception of the COMPASS model, all other models (Dreiding, UA, OPLS-AA, and HUANG) show a clear decreasing trend in thermal conductivity with increasing temperature, which aligns well with the experimental observations. Although the HUANG and UA force fields perform well in predicting most thermodynamic properties, they exhibit clear deviations in the predictions of temperature-dependent thermal conductivity: HUANG overestimates, while UA underestimates the experimental measurements.

#### Viscosity

**Figure 6:**
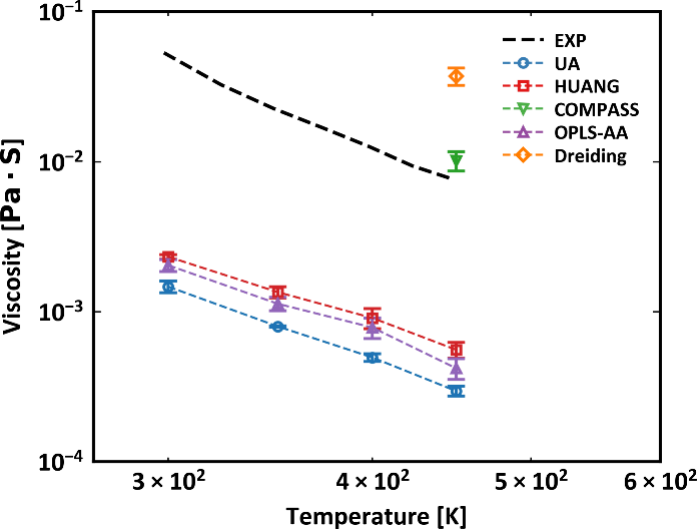
Viscosity as a function of temperature of PDMS simulated in log scale with 11 monomers.

We employed a PDMS system consisting of 100 chains, each with 11 monomers, for viscosity calculations at varying temperatures. As seen in Figure 6, the viscosity of these short-chain PDMS systems increases as the temperature decreases, following the Arrhenius-like behaviors in the melt state. Compared with the experimental measurements, our simulation results exhibit qualitatively consistent trends while predict significantly lower viscosities. This is primarily because the degree of polymerization in the simulated system is smaller than that in the experiments. It should be noted that we are not aiming for a quantitative comparison between simulations and experiments. Due to the constraints on the spatiotemporal scale accessible by atomistic MD, simulating PDMS systems with a high molecular weight, close to those used in experiments, presents significant challenges with current computational resources. Among the force fields that have been benchmarked, the two all-atom models, namely HUANG and OPLS-AA, as well as the UA model, are observed to predict lower viscosities, which aligns with expectations of the molecular weight dependence for polymer viscosity. Furthermore, the viscosity data for the COMPASS and Dreiding models are presented only at the highest temperature (*T* = 450 K) due to slow dynamics at lower temperatures, which impeded the efficient equilibration of the system. These results indicate that the predictions from the Dreiding and COMPASS universal force fields are considerably more viscous than experiments, considering the much lower molecular weights used in simulations compared with those in experiments.

## Discussion

**Figure 7:**
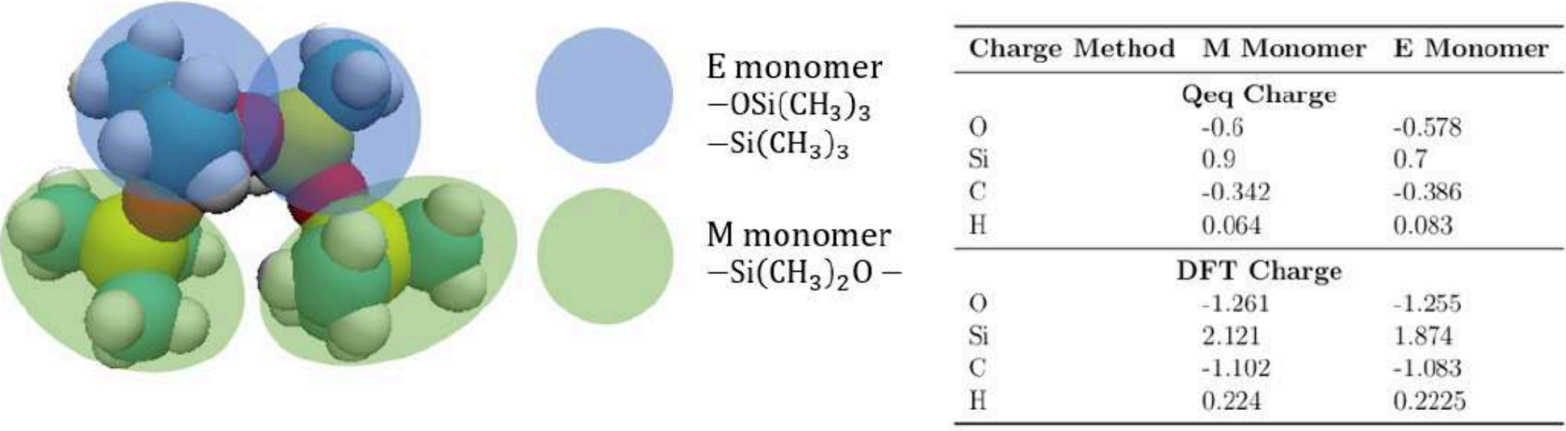
Illustration of the assignment of optimized point charges for oxygen, silicon, carbon, and hydrogen atoms of PDMS using QEq and DFT methods. E monomer is the monomer at the chain ends, and M monomer is the monomer within the chain.

**Figure 8:**
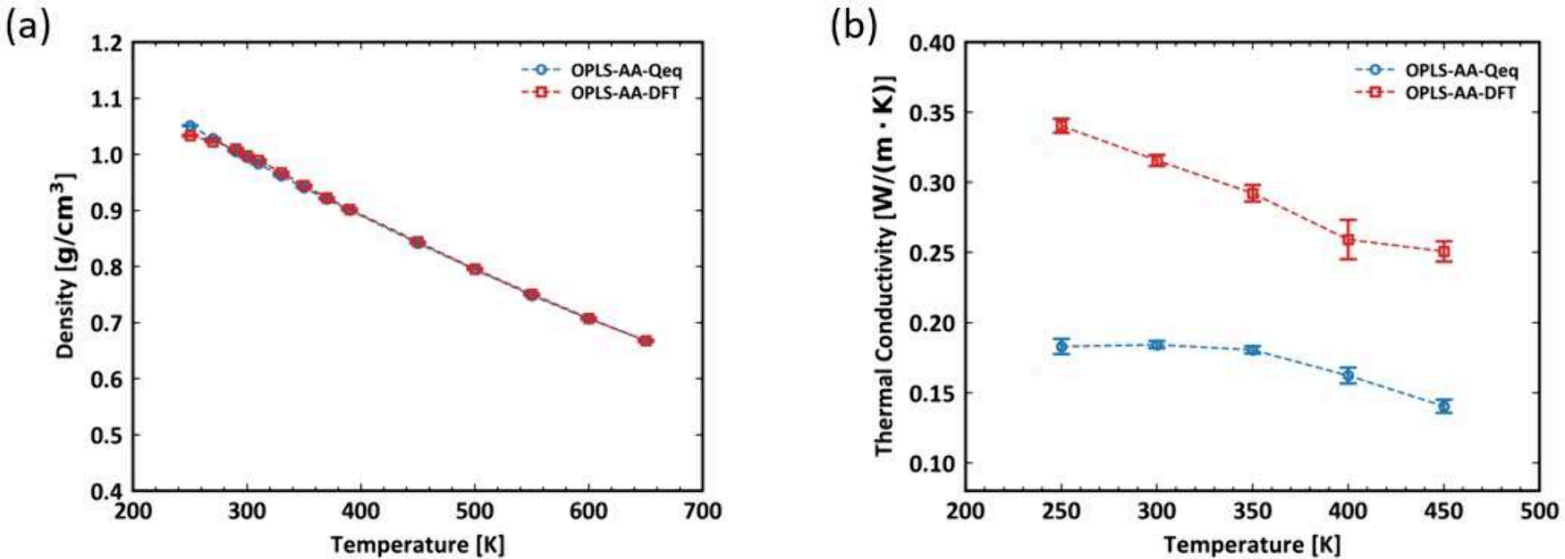
Comparison of density and thermal conductivity simulation with different charge methods: (a) density as a function of temperature of PDMS simulated; (b) thermal conductivity as a function of temperature of PDMS simulated.

We begin the discussion by examining the influence of atomic charges in PDMS systems on the prediction of various properties. In molecular dynamics (MD) simulations, atomic charges are essential for accurately modeling electrostatic interactions, which have a significant impact on simulation outcomes. While the charges in the original OPLS force field were empirically derived, primarily through fitting to reproduce the properties of organic liquids, several rigorous methods for charge determination have since been developed. Among the most widely used approaches are those based on the Charge Equilibration (Qeq) method and electronic structure calculations such as Density Functional Theory (DFT). In this study, we reparametrized the model using both the Natural Population Analysis (NPA) method, derived from DFT-optimized geometries, and Qeq techniques, as the original OPLS-AA force field lacked charge data for siloxane systems. The details on the DFT settings are provided in the Supporting Information. These two models are referred to as OPLS-AA-DFT and OPLS-AA-Qeq, respectively, to explicitly indicate the underlying methods used for charge parametrization. The derived charges are presented in Figure 7, highlighting the noticeable differences between these methods. We evaluate the performance based on two specific properties, namely, density and thermal conductivity, using both sets of charges, while keeping all other bonded and nonbonded interactions the same. The results are presented in Figure 8. As shown in Figure 8(a), the densities are almost identical. However, Figure 8(b) shows that the thermal conductivity simulated with DFT charges is nearly twice that obtained from Qeq charges, with a greater slope. Moreover, the thermal conductivity determined using models with DFT-derived partial charges shows greater discrepancies compared to experimental results. Therefore, the Qeq charges are employed for the rest of the simulations. The inaccuracies observed in simulations using DFT-derived charges likely arise from their inconsistency with the force field parametrization method, where the original OPLS force field employs charges empirically fitted to experimental data rather than derived from ab initio calculations.^33^ Moreover, the charges in OPLS-AA are parametrized to be consistent with those in the original OPLS-UA, where +0.06 e was added to alkene hydrogen atoms, closely resembling the values obtained in this study using the Qeq method. In contrast, charges derived from DFT-optimized geometries typically exhibit larger magnitudes, as seen in the table below, leading to a mismatch with the charge distribution assumed in OPLS-AA. This inconsistency in charge assignment can result in an inaccurate representation of electrostatic interactions, thereby affecting the reliability of predictions for energy transport properties such as thermal conductivity.

In benchmarking various force fields for simulating polydimethylsiloxane (PDMS), several models exhibit outlier behavior when predicting material properties, as determined through comparisons with experimental measurements. In Table 4, we present a summary of the performance of these force fields, using the root-mean-square error (RMSE) as the metric, to predict the selected properties measured by the experiments in this study. We find that a force field that accurately predicts one property does not necessarily guarantee accurate predictions for other properties. For example, the improved Class II model developed by Huang et al. provides density predictions in good agreement with experimental data over a wide temperature range but significantly overestimates thermal conductivity. A second observation is that, for universal force fields, including specific chemical environments in the parametrization process does not always lead to better predictions. For instance, although the COMPASS force field incorporates parametrization of the silicon environment, its predictions for PDMS systems at varying temperatures are not consistently superior to those of models that do not include silicon-specific optimizations. Furthermore, the Class II force field does not always outperform the Class I force field in terms of predictive accuracy. As shown in Table 4, compared with the Class II COMPASS force field, the Class I OPLS-AA model exhibits a generally comparable or even lower error for the properties tested in this study.

**Table 4:** Root Mean Square Error (RMSE) for the Simulation Results with Various Force Fields Compared with Experimental Measurements

Model/RMSE	Density [g/cm^3^]	Heat Capacity [J/(g ·K)]	Thermal Conductivity [W/(m·K)]
UA	0.0523	12.4543	0.0308
HUANG	**0.0101**	**2.0657**	0.0438
COMPASS	0.1781	2.5284	0.0360
OPLS-AA	0.0414	2.1320	0.0340
Dreiding	0.1337	2.1338	**0.0208**

This benchmark study offers valuable insights on the improvement of force fields for simulating specific systems. To enhance the accuracy of PDMS simulations, Huang et al. employed high-level quantum calculations to optimize atomic charges and bonded interaction parameters, while refining nonbonded interactions by fitting experimental data for density and vaporization enthalpy of short PDMS oligomers. This combined bottom-up (quantum-based) and top-down approach (experimentally driven) confirms an effective strategy for developing accurate and transferable force fields for specific systems. Additionally, the OPLS-AA force field, with optimized atomic charges for the siloxane environment, demonstrates sufficient performance in predicting both the thermodynamic and transport properties. This underscores the critical role of atomic charge optimization in enhancing both the accuracy and transferability of force fields. Recent simulations further support the idea that optimizing atomic charges for specific systems is a promising approach to improving force field reliability.^34,35^

## Conclusion

In this study, we conduct a systematic benchmark of five different force fields, UA, HUANG’s Class II model, COMPASS, OPLS-AA, and Dreiding, for their ability to simulate the thermodynamic and transport behaviors of PDMS systems. Our findings highlight the varied performance of these models across different properties, emphasizing the importance of selecting the appropriate force field for a given application. Specifically, the united-atom (UA) model, although computationally efficient, significantly underperforms compared to the all-atom (AA) models. HUANG’s Class II model, however, provides a closer match to experimental data, especially at 300 K, making it a more reliable choice for density predictions. Nonetheless, slight deviations are observed at higher temperatures, suggesting that further refinements may be necessary for broader temperature ranges. The universal force fields, such as OPLS-AA, COMPASS, and Dreiding, are widely utilized for their flexibility across diverse molecular systems. While they show promise in accurately simulating diverse polysiloxane materials, certain limitations are also revealed. For instance, COMPASS consistently overestimates densities and its slower dynamics at lower temperatures affects the accuracy of viscosity predictions. Similarly, Dreiding, while offering reasonable structural predictions, also exhibits more viscous behaviors compared to experimental measurements.

One of the key challenges identified in this study is the transferability of force fields. Although many of these models are optimized for specific properties, such as density and heat of vaporization, their ability to accurately predict other critical properties, such as viscosity and thermal conductivity, remains limited. This issue highlights the need for force-field refinement or the development of improved models that can more reliably capture both thermodynamic and transport properties across a wider range of conditions. Among the five models benchmarked in this study, while none of them achieved a perfect match with the experimental data in all properties, the HUANG Class II model emerged as one of the most reliable options to simulate PDMS, especially in predicting density, as evidenced by the lower RMSE values presented in the Table 4. Moreover, for simulations requiring the inclusion of a broader range of chemical species, practitioners may consider using the OPLS-AA force field as a viable alternative.

Future work should focus on addressing the identified limitations, such as optimizing point charges and improving model prediction capability on dynamical properties, to enhance the accuracy and transferability in force-field-based MD simulations of PDMS and its derivatives with side-group modifications. Such improvements are essential for advancing the computational modeling of polysiloxane polymers and other complex systems. Recent advances in machine learning force fields (MLFF) present a promising approach to address these challenges.^36−38^ MLFFs aim to reconcile the precision of ab initio methods with the computational efficiency of classical force fields by learning statistical relationships between chemical structures/configurations and potential energy, without relying on predefined assumptions about chemical bonds or specific interactions. By leveraging extensive data sets and refining parameters beyond traditional approaches, MLFFs are expected to enable more accurate and transferable predictions from MD simulations.^38,39^ In addition, due to the significant computational cost of atomistic models, our benchmarked systems are restricted to short-chain configurations. Employing systematic coarse-graining techniques might enable simulations on a larger spatiotemporal scale to capture the molecular weight dependence of PDMS systems.^40,41^ To access systems with even higher molecular weight, e.g., > 10 *M*_*e*_, where *M*_*e*_ is the entanglement molecular weight, the atomistic models benchmarked in this work can be utilized as inputs for constructing multichain slip-spring models within a bottom-up multiscale framework, as demonstrated in a series of recent studies.^42−44^ Overall, this study provides a comprehensive assessment of force-field performance for polysiloxanes, contributing to the integration of computational modeling with experimental observations. The findings in our study are anticipated to inform the selection and development of force fields, advancing the efficient computational design and optimization of polysiloxane materials for industrial, medical, and technological applications.

## Supporting Information Available

Details about the molecular dynamics simulations of PDMS: force-field parameters of OPLS-AA, Dreiding, COMPASS, HUANG, UA; convergence of the autocorrelation of chain end-to-end vectors for viscosity calculations; comparison between computational speed of various force fields; details on experimental measurements (PDF)

## References

[ref1] Lötters J. C.; Olthuis W.; Veltink P. H.; Bergveld P. The mechanical properties of the rubber elastic polymer polydimethylsiloxane for sensor applications. J. Micromech. Microeng. 1997, 7, 145–147. 10.1088/0960-1317/7/3/017.

[ref2] Mata A.; Fleischman A. J.; Roy S. Characterization of Polydimethylsiloxane (PDMS) Properties for Biomedical Micro/Nanosystems. Biomed Microdevices 2005, 7, 281–293. 10.1007/s10544-005-6070-2. 16404506

[ref3] Cho S.; Andersson H.; White S.; Sottos N.; Braun P. Polydimethylsiloxane-Based Self-Healing Materials. Adv. Mater. 2006, 18, 997–1000. 10.1002/adma.200501814.

[ref4] Schneider F.; Draheim J.; Kamberger R.; Wallrabe U. Process and material properties of polydimethylsiloxane (PDMS) for Optical MEMS. Sensors and Actuators A: Physical 2009, 151, 95–99. 10.1016/j.sna.2009.01.026.

[ref5] Seethapathy S.; Górecki T. Applications of polydimethylsiloxane in analytical chemistry: A review. Anal. Chim. Acta 2012, 750, 48–62. 10.1016/j.aca.2012.05.004. 23062428

[ref6] Halldorsson S.; Lucumi E.; Gómez-Sjöberg R.; Fleming R. M. Advantages and challenges of microfluidic cell culture in polydimethylsiloxane devices. Biosens. Bioelectron. 2015, 63, 218–231. 10.1016/j.bios.2014.07.029. 25105943

[ref7] Sok R. M.; Berendsen H. J. C.; Van Gunsteren W. F. Molecular Dynamics Simulation of the Transport of Small Molecules across a Polymer Membrane. J. Chem. Phys. 1992, 96, 4699–4704. 10.1063/1.462806.

[ref8] Heine D. R.; Grest G. S.; Lorenz C. D.; Tsige M.; Stevens M. J. Atomistic Simulations of End-Linked Poly(dimethylsiloxane) Networks: Structure and Relaxation. Macromolecules 2004, 37, 3857–3864. 10.1021/ma035760j.

[ref9] Luo T.; Esfarjani K.; Shiomi J.; Henry A.; Chen G. Molecular dynamics simulation of thermal energy transport in polydimethylsiloxane. J. Appl. Phys. 2011, 109, 074321 10.1063/1.3569862.

[ref10] Chang K.-S.; Chung Y.-C.; Yang T.-H.; Lue S. J.; Tung K.-L.; Lin Y.-F. Free volume and alcohol transport properties of PDMS membranes: Insights of nano-structure and interfacial affinity from molecular modeling. J. Membr. Sci. 2012, 417–418, 119–130. 10.1016/j.memsci.2012.06.019.

[ref11] Lou W.; Xie C.; Guan X. Understanding radiation-thermal aging of polydimethylsiloxane rubber through molecular dynamics simulation. npj Mater. Degrad 2022, 6, 84 10.1038/s41529-022-00299-1.

[ref12] Zhang W.; Hu Z.; Lu Y.; Zhou T.; Zhang H.; Zhao X.; Liu L.; Zhang L.; Gao Y. Molecular Dynamics Simulation on the Heat Transfer in the Cross-Linked Poly(dimethylsiloxane). J. Phys. Chem. B 2023, 127, 10243–10251. 10.1021/acs.jpcb.3c06476. 37975617

[ref13] Sun H. COMPASS: An Ab Initio Force-Field Optimized for Condensed-Phase ApplicationsOverview with Details on Alkane and Benzene Compounds. J. Phys. Chem. B 1998, 102, 7338–7364. 10.1021/jp980939v.

[ref14] Huang H.; Cao F.; Wu L.; Sun H. All-Atom and Coarse-Grained Force Fields for Polydimethylsiloxane. Mol. Simul. 2017, 43, 1513–1522. 10.1080/08927022.2017.1328597.

[ref15] Frischknecht A. L.; Curro J. G. Improved United Atom Force Field for Poly(dimethylsiloxane). Macromolecules 2003, 36, 2122–2129. 10.1021/ma025763g.

[ref16] Jorge M.; Milne A. W.; Barrera M. C.; Gomes J. R. New Force-Field for Organosilicon Molecules in the Liquid Phase. ACS Physical Chemistry Au 2021, 1, 54–69. 10.1021/acsphyschemau.1c00014. 34939073 PMC8679648

[ref17] Sun H. Ab initio calculations and force field development for computer simulation of polysilanes. Macromolecules 1995, 28, 701–712. 10.1021/ma00107a006.

[ref18] Smith J. S.; Borodin O.; Smith G. D. A Quantum Chemistry Based Force Field for Poly(dimethylsiloxane). J. Phys. Chem. B 2004, 108, 20340–20350. 10.1021/jp047434r.

[ref19] Jorgensen W. L.; Maxwell D. S.; Tirado-Rives J. Development and Testing of the OPLS All-Atom Force Field on Conformational Energetics and Properties of Organic Liquids. J. Am. Chem. Soc. 1996, 118, 11225–11236. 10.1021/ja9621760.

[ref20] Caleman C.; Van Maaren P. J.; Hong M.; Hub J. S.; Costa L. T.; Van Der Spoel D. Force Field Benchmark of Organic Liquids: Density, Enthalpy of Vaporization, Heat Capacities, Surface Tension, Isothermal Compressibility, Volumetric Expansion Coefficient, and Dielectric Constant. J. Chem. Theory Comput. 2012, 8, 61–74. 10.1021/ct200731v. 22241968 PMC3254193

[ref21] Mayo S. L.; Olafson B. D.; Goddard W. A. DREIDING: a generic force field for molecular simulations. J. Phys. Chem. 1990, 94, 8897–8909. 10.1021/j100389a010.

[ref22] Xiang Z. PDMS Benchmark. https://github.com/COMO-lab/PDMS_benchmark, 2024; Accessed: January 11, 2025.

[ref23] Rappe A. K.; Goddard W. A. Charge equilibration for molecular dynamics simulations. J. Phys. Chem. 1991, 95, 3358–3363. 10.1021/j100161a070.

[ref24] Fonseca Guerra C.; Handgraaf J.; Baerends E. J.; Bickelhaupt F. M. Voronoi deformation density (VDD) charges: Assessment of the Mulliken, Bader, Hirshfeld, Weinhold, and VDD methods for charge analysis. J. Comput. Chem. 2004, 25, 189–210. 10.1002/jcc.10351. 14648618

[ref25] Jewett A. I.; Stelter D.; Lambert J.; Saladi S. M.; Roscioni O. M.; Ricci M.; Autin L.; Maritan M.; Bashusqeh S. M.; Keyes T.; et al. Moltemplate: A Tool for Coarse-Grained Modeling of Complex Biological Matter and Soft Condensed Matter Physics. J. Mol. Biol. 2021, 433, 166841 10.1016/j.jmb.2021.166841. 33539886 PMC8119336

[ref26] Hockney R. W.; Eastwood J. W. Computer simulation using particles, special student ed.; A. Hilger: Bristol [England], Philadelphia, 1988.

[ref27] Thompson A. P.; Aktulga H. M.; Berger R.; Bolintineanu D. S.; Brown W. M.; Crozier P. S.; in ’t Veld P. J.; Kohlmeyer A.; Moore S. G.; Nguyen T. D.; et al. LAMMPS - a flexible simulation tool for particle-based materials modeling at the atomic, meso, and continuum scales. Comput. Phys. Commun. 2022, 271, 108171 10.1016/j.cpc.2021.108171.

[ref28] Martínez L.; Andrade R.; Birgin E. G.; Martínez J. M. P < span style = ”font-variant:small-caps;”> ACKMOL < /span > : A package for building initial configurations for molecular dynamics simulations. J. Comput. Chem. 2009, 30, 2157–2164. 10.1002/jcc.21224. 19229944

[ref29] Kubo R. Statistical-Mechanical Theory of Irreversible Processes. I. General Theory and Simple Applications to Magnetic and Conduction Problems. J. Phys. Soc. Jpn. 1957, 12, 570–586. 10.1143/JPSJ.12.570.

[ref30] Hoover W. G.; Evans D. J.; Hickman R. B.; Ladd A. J. C.; Ashurst W. T.; Moran B. Lennard-Jones triple-point bulk and shear viscosities. Green–Kubo theory, Hamiltonian mechanics, and nonequilibrium molecular dynamics. Phys. Rev. A 1980, 22, 1690–1697. 10.1103/PhysRevA.22.1690.

[ref31] Zhang Y.; Otani A.; Maginn E. J. Reliable Viscosity Calculation from Equilibrium Molecular Dynamics Simulations: A Time Decomposition Method. J. Chem. Theory Comput. 2015, 11, 3537–3546. 10.1021/acs.jctc.5b00351. 26574439

[ref32] Shih H.; Flory P. J. Equation-of-State Parameters for Poly(Dimethylsiloxane). Macromolecules 1972, 5, 758–761. 10.1021/ma60030a018.

[ref33] Jorgensen W. L.; Maxwell D. S.; Tirado-Rives J. Development and Testing of the OPLS All-Atom Force Field on Conformational Energetics and Properties of Organic Liquids. J. Am. Chem. Soc. 1996, 118, 11225–11236. 10.1021/ja9621760.

[ref34] Siu S. W. I.; Pluhackova K.; Böckmann R. A. Optimization of the OPLS-AA Force Field for Long Hydrocarbons. J. Chem. Theory Comput. 2012, 8, 1459–1470. 10.1021/ct200908r. 26596756

[ref35] Thaler S.; Mayr F.; Thomas S.; Gagliardi A.; Zavadlav J. Active learning graph neural networks for partial charge prediction of metal-organic frameworks via dropout Monte Carlo. npj Comput. Mater. 2024, 10, 86 10.1038/s41524-024-01277-8.

[ref36] Unke O. T.; Chmiela S.; Sauceda H. E.; Gastegger M.; Poltavsky I.; Schütt K. T.; Tkatchenko A.; Müller K.-R. Machine Learning Force Fields. Chem. Rev. 2021, 121, 10142–10186. 10.1021/acs.chemrev.0c01111. 33705118 PMC8391964

[ref37] Fu X.; Wu Z.; Wang W.; Xie T.; Keten S.; Gomez-Bombarelli R.; Jaakkola T. Forces Are Not Enough: Benchmark and Critical Evaluation for Machine Learning Force Fields with Molecular Simulations. arXiv:2210.07237 2022, 10.48550/arXiv.2210.07237.

[ref38] Kovacs D. P.; Moore J. H.; Browning N. J.; Batatia I.; Horton J. T.; Kapil V.; Witt W. C.; Magd I.-B. MACE-OFF23: Transferable Machine Learning Force Fields for Organic Molecules. arXiv:2312.15211,2023, 10.48550/arXiv.2312.15211. PMC12123624 40387214

[ref39] Wang T.; He X.; Li M.; Li Y.; Bi R.; Wang Y.; Cheng C.; Shen X.; Meng J.; Zhang H.; et al. Ab initio characterization of protein molecular dynamics with AI2BMD. Nature 2024, 635, 1019–1027. 10.1038/s41586-024-08127-z. 39506110 PMC11602711

[ref40] Noid W. G. Perspective: Advances, Challenges, and Insight for Predictive Coarse-Grained Models. J. Phys. Chem. B 2023, 127, 4174–4207. 10.1021/acs.jpcb.2c08731. 37149781

[ref41] Wu Z.; Zhou T. Structural Coarse-Graining via Multiobjective Optimization with Differentiable Simulation. J. Chem. Theory Comput. 2024, 20, 2605–2617. 10.1021/acs.jctc.3c01348. 38483262

[ref42] Wu Z.; Milano G.; Müller-Plathe F. Combination of Hybrid Particle-Field Molecular Dynamics and Slip-Springs for the Efficient Simulation of Coarse-Grained Polymer Models: Static and Dynamic Properties of Polystyrene Melts. J. Chem. Theory Comput. 2021, 17, 474–487. 10.1021/acs.jctc.0c00954. 33275441

[ref43] Wu Z.; Müller-Plathe F. Slip-Spring Hybrid Particle-Field Molecular Dynamics for Coarse-Graining Branched Polymer Melts: Polystyrene Melts as an Example. J. Chem. Theory Comput. 2022, 18, 3814–3828. 10.1021/acs.jctc.2c00107. 35617016

[ref44] Liang H.; Yoshimoto K.; Gil P.; Kitabata M.; Yamamoto U.; De Pablo J. J. Bottom-Up Multiscale Approach to Estimate Viscoelastic Properties of Entangled Polymer Melts with High Glass Transition Temperature. Macromolecules 2022, 55, 3159–3165. 10.1021/acs.macromol.1c02044.

