# Supplementary material for: Force-Field Benchmark for Polydimethylsiloxane: Density, Heat Capacity, Isothermal Compressibility, Viscosity and Thermal Conductivity
Source: J Phys Chem B. 2025 Feb 3;129(6):1864–73. doi: 10.1021/acs.jpcb.4c08471 (PMC11831649; doi:10.1021/acs.jpcb.4c08471)
Supplement: Supplementary file 1 — jp4c08471_si_001.pdf [file jp4c08471_si_001.pdf]

Supporting Information: Force-Field Benchmark for Polydimethylsiloxane: Density, Heat Capacity, Isothermal Compressibility, Viscosity and Thermal Conductivity

Zhirui Xiang,[†] Chao Gao,[‡] Teng Long,[¶] Lifeng Ding,[†] Tianhang Zhou,[§] and Zhenghao Wu[*,†]

†*Department of Chemistry and Materials Science, Xi'an Jiaotong-Liverpool University, Suzhou 215123, Jiangsu, P. R. China*

‡*Aerospace Research Institute of Materials & Processing Technology, Beijing, 100076, P. R. China;*

¶*School of Materials Science & Engineering, Shandong University, Jingshi Road, 17923, Jinan, Shandong, China*

§*College of Carbon Neutrality Future Technology, State Key Laboratory of Heavy Oil Processing, China University of Petroleum (Beijing), Beijing 102249, P. R. China*

E-mail: zhenghao.wu@xjtlu.edu.cn

Details of Force-Fields

Improved UA model

Initially, single-chain configurations, including coordinates, atom indices, and bond indices, were obtained using Avogadro Software,[1] with hydrogen atoms omitted during modelling. Packmol was then employed to randomly place a specified number of chains within a simulation box. Python scripts were subsequently used to generate system topologies, including atoms, bonds, angles, and dihedrals. Simulation parameters and partial charges were sourced from Frischknecht and Curro.[2]

Table S1: Parameters for United Atom force field model

Charge					
O	-0.3				
Si	0.3				
C	0				
pair_style hybrid lj/class2/coul/cut 10.0 lj/cut/coul/cut 12.0					
pair_coeff	O	O	lj/class2/coul/cut	0.08	3.3
pair_coeff	O	Si	lj/class2/coul/cut	0.0772	3.94
pair_coeff	Si	Si	lj/class2/coul/cut	0.131	4.29
pair_coeff	O	C	lj/cut/coul/cut	0.1247	3.38
pair_coeff	Si	C	lj/cut/coul/cut	0.1596	3.83
pair_coeff	C	C	lj/cut/coul/cut	0.1944	3.73
bond_style harmonic					
bond_coeff	Si-O	350.12	1.64		
bond_coeff	Si-C	189.65	1.9		
angle_style harmonic					
angle_coeff	C-Si-C	49.97	109.24		
angle_coeff	C-Si-O	49.97	110.69		
angle_coeff	O-Si-O	94.5	107.82		
angle_coeff	Si-O-Si	14.14	146.46		
dihedral_style harmonic					
dihedral_coeff	Si-O-Si-C	0.01	1	3	
dihedral_coeff	Si-O-Si-O	0.225	1	1	

Class2 Model from HUANG

The topology generation strategy mirrored that of the improved UA model, with the addition of hydrogen atoms on methyl groups to align with all-atom force field requirements. All

simulation parameters used in our study were derived from HUANG et al.[3]

Table S2: Parameters for HUANG's all-atom force field model

charge	inside monomer	end monomer				
O	-0.13133	-0.13456				
Si	0.17557	0.172334				
C	-0.05053	-0.05375				
H	0.00947	0.006244				
pair_style	lj/cut/coul/long	12				
pair_coeff	O	O	0.1135	3.2		
pair_coeff	Si	Si	0.1035	4.135		
pair_coeff	C	C	0.0492	3.695		
pair_coeff	H	H	0.0299	2.4335		
bond_style	class2					
bond_coeff	o_2-si_4	1.6818	241.59	-483.18	563.71	
bond_coeff	c_4-si_4	1.893	160.41	-320.82	374.29	
bond_coeff	c_4-h_1	1.0967	374.52	-749.04	873.88	
angle_style	class2					
angle_coeff	c_4-si_4-c_4	112.1	60.1	-0.2327	-0.00394793	
angle_coeff	c_4-si_4-o_2	109.47	69.01	-0.232	-0.00446903	
angle_coeff	h_1-c_4-h_1	106.77	42.51	-0.1216	-0.00271846	
angle_coeff	h_1-c_4-si_4	110.2	31.27	-0.1095	-0.00203264	
angle_coeff	o_2-si_4-o_2	109.41	81.05	-0.2716	-0.00524714	
angle_coeff	si_4-o_2-si_4	149.09	18.51	-0.3165	-0.0020083	
angle_coeff	h_c_h	bb	7.1081	1.0967	1.0967	
angle_coeff	o_si_o	bb	-85.9036	1.6818	1.6818	
angle_coeff	si_o_si	bb	23.6039	1.6818	1.6818	
angle_coeff	h_c_h	ba	17.836	17.836	1.0967	1.0967
angle_coeff	o_si_o	ba	-18.1072	-18.1072	1.6818	1.6818
angle_coeff	si_o_si	ba	42.3234	42.3234	1.6818	1.6818
dihedral_style	opls					
dihedral_coeff	c_4-si_4-c_4-h_1	0	0	0.15	0	
dihedral_coeff	c_4-si_4-o_2-si_4	0	0	0.06	0	
dihedral_coeff	h_1-c_4-si_4-o_2	0	0	0.15	0	
dihedral_coeff	o_2-si_4-o_2-si_4	0	0	0.2416	0	

COMPASS

In the preliminary stage, the monomer structure was generated using Avogadro Software.[1] Polymer chains were then constructed through the replication of monomers and chains, followed by energy minimization using COMPASS parameters integrated into the Moltemplate

package.[?] The resulting configuration was exported into LAMMPS data file format for subsequent simulations.

Table S3: Parameters for COMPASS force field model

Charge						
O	-0.89					
Si	1.255					
C	-0.588					
H	0.106					
pair_style	lj/class2/coul/long 10					
pair_coeff	H	H	0.0230	2.878		
pair_coeff	O	O	0.0800	3.3		
pair_coeff	C	C	0.0620	3.854		
pair_coeff	Si	Si	0.1310	4.29		
bond_style	class2					
bond_coeff	o_si	1.64	350.1232	-517.3424	673.7067	
bond_coeff	c_h	1.101	345	-691.89	844.6	
bond_coeff	si_c	1.8995	189.6536	-279.421	307.5135	
angle_style	class2					
angle_coeff	c_si_o		113.1855	36.2069	-20.3939	20.0172
angle_coeff	c_si_o	bb	3.7419	1.8995	1.8995	
angle_coeff	c_si_o	ba	18.5805	18.5805	1.8995	1.8995
angle_coeff	si_c_h		112.0355	28.7721	-13.9523	0
angle_coeff	si_c_h	bb	1.6561	1.101	1.8995	
angle_coeff	si_c_h	ba	16.6908	18.2764	1.101	1.8995
angle_coeff	o_si_o		110.7	70.3069	-6.9375	0
angle_coeff	o_si_o	bb	41.1143	1.64	1.64	
angle_coeff	o_si_o	ba	23.438	23.438	1.64	1.64
angle_coeff	c_si_c		114.906	23.0218	-31.3993	24.9814
angle_coeff	c_si_c	bb	5.4896	1.8995	1.64	
angle_coeff	c_si_c	ba	6.4278	20.5669	1.8995	1.64
angle_coeff	h_c_h		107.66	39.641	-12.921	-2.4318
angle_coeff	h_c_h	bb	5.3316	1.101	1.101	
angle_coeff	h_c_h	ba	18.103	18.103	1.101	1.101
angle_coeff	si_o_si		159	8.5	-13.4188	-4.1785
angle_coeff	si_o_si	bb	41.1143	1.64	1.64	
angle_coeff	si_o_si	ba	28.6686	28.6686	1.64	1.64

dihedral_style	class2									
dihedral_coeff	o_si_c_h		0	0	0	0	-0.0657	0		
dihedral_coeff	o_si_c_h	mbt	0	0	0	1.8995				
dihedral_coeff	o_si_c_h	ebt	0	0	0	0	0	0	1.101	1.64
dihedral_coeff	o_si_c_h	at	0	0	0	0	0	0	112.0355	114.906
dihedral_coeff	o_si_c_h	aat	0	112.0355	114.906					
dihedral_coeff	o_si_c_h	bb13	0	1.101	1.64					
dihedral_coeff	c_si_c_h		0	0	0	0	-0.0657	0		
dihedral_coeff	c_si_c_h	mbt	0	0	0	1.8995				
dihedral_coeff	c_si_c_h	ebt	0	0	0	0	0	0	1.101	1.8995
dihedral_coeff	c_si_c_h	at	0	0	0	0	0	0	112.0355	113.1855
dihedral_coeff	c_si_c_h	aat	0	112.0355	113.1855					
dihedral_coeff	c_si_c_h	bb13	0	1.101	1.8995					
dihedral_coeff	si_o_si_o		-0.225	0	0	0	-0.01	0		
dihedral_coeff	si_o_si_o	mbt	0	0	0	1.64				
dihedral_coeff	si_o_si_o	ebt	0	0	0	0	0	0	1.64	1.64
dihedral_coeff	si_o_si_o	at	0	0	0	0	0	0	110.7	159
dihedral_coeff	si_o_si_o	aat	0	110.7	159					
dihedral_coeff	si_o_si_o	bb13	0	1.64	1.64					
dihedral_coeff	c_si_o_si		0	0	0	0	-0.01	0		
dihedral_coeff	c_si_o_si	mbt	0	0	0	1.64				
dihedral_coeff	c_si_o_si	ebt	0	0	0	0	0	0	1.8995	1.64
dihedral_coeff	c_si_o_si	at	0	0	0	0	0	0	114.906	159
dihedral_coeff	c_si_o_si	aat	0	114.906	159					
dihedral_coeff	c_si_o_si	bb13	0	1.8995	1.64					
improper_style	class2									
dihedral_coeff	si_c_c_h		0	0	0	0	0	0		
dihedral_coeff	si_c_c_h	mbt	1.3445	3.5515	-4.9202	1.375				
dihedral_coeff	si_c_c_h	ebt	0.1928	1.3187	0.8599	0.0004	-1.0975	0.4831	1.514	1.42
dihedral_coeff	si_c_c_h	at	0.9701	-2.5169	1.7195	0.8831	-0.8203	0.2405	100.3182	109
dihedral_coeff	si_c_c_h	aat	-12.207	100.3182	109					
dihedral_coeff	si_c_c_h	bb13	0	1.514	1.42					
dihedral_coeff	h_c_h_h		0	0	0	0	-0.0657	0		
dihedral_coeff	h_c_h_h	mbt	0	0	0	2.3384				
dihedral_coeff	h_c_h_h	ebt	0	0	0	0	0	0	2.3384	1.8995
dihedral_coeff	h_c_h_h	at	0	0	0	0	0	0	114.2676	113
dihedral_coeff	h_c_h_h	aat	0	114.2676	113					
dihedral_coeff	h_c_h_h	bb13	0	2.3384	1.8995					

OPLS-AA

The OPLS-AA modelling process followed a similar methodology to that of COMPASS. Force field parameters were obtained from the Moltemplate package.[4,?] However, we parameterized the partial charges of PDMS using two strategies. NPA charges were derived from DFT calculations of 3-5 monomers in Gaussian software, while Qeq formal charges were assigned using the charge equilibration method[5] (Qeq) in Materials Studio 7.0. These charges were then employed in molecular dynamics simulations.

Table S4: Parameters for OPLS-AA force field model

charge	inside monomer	end monomer			
O	-0.6	-0.578			
Si	0.9	0.7			
C	-0.342	-0.386			
H	0.064	0.083			
pair_style	lj/cut/coul/long 11 11				
pair_coeff	O	O	0.14	2.9	
pair_coeff	Si	Si	0.1	4	
pair_coeff	C	C	0.066	3.5	
pair_coeff	H	H	0.03	2.5	
bond_style	harmonic				
bond_coeff	o_si	374	1.64		
bond_coeff	si_c	187	1.86		
bond_coeff	c_h	340	1.09		
angle_style	harmonic				
angle_coeff	h_c_h	33	107.8		
angle_coeff	si_c_h	35	109.5		
angle_coeff	si_o_si	20	145		
angle_coeff	c_si_c	60	110		
angle_coeff	c_si_o	60	100		
angle_coeff	o_si_o	60	110		
dihedral_style	opls				
dihedral_coeff	c_si_c_h	0	0	0.18	0
dihedral_coeff	o_si_c_h	0	0	0	0
dihedral_coeff	si_o_si_o	0	0	0	0
dihedral_coeff	c_si_o_si	0	0	0	0

Dreiding

The Dreiding modelling process also followed a methodology similar to that of COMPASS. Force field parameters were obtained from the Moltemplate package, and the same partial charges from the Qeq method used in the OPLS-AA model were employed in molecular dynamics simulations. For the sake of computational efficiency, long-range electrostatic interactions were handled using the Particle-Particle Particle-Mesh (PPPM) method with an accuracy threshold of 0.0001.

Table S5: Parameters for Dreiding force field model

charge	inside monomer	end monomer			
O	-0.6	-0.578	35		
Si	0.9	0.7	58		
C	-0.342	-0.386	3		
H	0.064	0.083	1		
pair_style	hybrid/overlay	hbond/dreiding/lj	4 6 6.5 90	lj/cut/coul/long	10
pair_coeff	H	C	lj/cut/coul/long	0.03801999	3.1597055
pair_coeff	H	H	lj/cut/coul/long	0.0152	2.846421
pair_coeff	C	Si	lj/cut/coul/long	0.17170032	3.638564
pair_coeff	C	O	lj/cut/coul/long	0.09539953	3.2530715
pair_coeff	H	O	lj/cut/coul/long	0.03813974	2.939787
pair_coeff	H	Si	lj/cut/coul/long	0.06864401	3.3252795
pair_coeff	O	Si	lj/cut/coul/long	0.17224111	3.4186455
pair_coeff	C	Si	lj/cut/coul/long	0.1744477	3.531656
pair_coeff	C	C	lj/cut/coul/long	0.0951	3.47299
pair_coeff	O	O	lj/cut/coul/long	0.0957	3.033153
pair_coeff	Si	Si	lj/cut/coul/long	0.31	3.804138
pair_coeff	H	Si	lj/cut/coul/long	0.06974238	3.2183715
pair_coeff	O	Si	lj/cut/coul/long	0.17499714	3.3117375
bond_style	harmonic				
bond_coeff	o_si	350	1.587		
bond_coeff	si_c	350	1.697		
bond_coeff	c_h	350	1.09		
angle_style	harmonic				
angle_coeff	si_c_h	50	109.471		
angle_coeff	si_o_si	50	104.51		
angle_coeff	o_si_c	50	109.471		
dihedral_style	charmm				
dihedral_coeff	o_si_c_h	0.1111	3	720	0
dihedral_coeff	si_o_si_o	0.3333	3	720	0

Partial charge calculation with DFT optimized geometry

To derive the partial charges, Density Functional Theory (DFT) calculations were conducted using the Gaussian 09 software package.[6] Geometry optimizations and frequency calculations were performed for PDMS structures containing 3, 4, and 5 monomer units. The B3LYP functional with the 6-311G(d,p) basis set was employed for these calculations. Natural Population Analysis (NPA) was used to calculate partial atomic charges from the optimized structures. The resulting charges served as the basis for force field parameterization.

Details of simulation

System Stablization From Time-series Plot of Energy

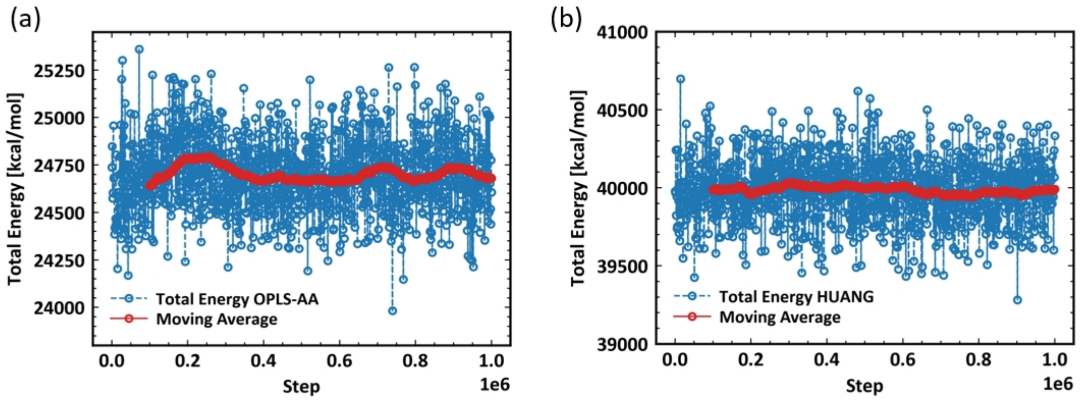

Figure S1: Total energy of the simulation system and moving average after equilibration at T=300 K, with window size=100 (a) OPLS-AA force field (b) HUANG force field

Autocorrelation of Chain End-to-End Vectors

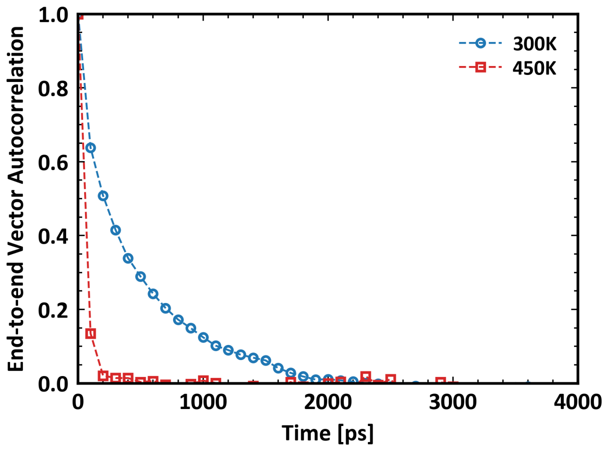

Figure S2: Autocorrelation function of chain end-to-end vectors as a function of time for PDMS melt with 11 monomers at high temperature (T=450 K) and low temperature (T=300 K).

System Size Effect Evaluation

The system size evaluation aims to confirm that the system is sufficiently large and that size-related effects do not significantly influence the results. To achieve this, simulations for viscosity and thermal conductivity calculation were conducted with varying system sizes. For viscosity, simulations were performed using a system of 11 monomers with 50, 100, and 200 chains, employing the UA model at a temperature of 450 K. For thermal conductivity, a system of 50 monomers with 64, 125, and 216 chains was used, with simulations performed at 300 K using the COMPASS force field.

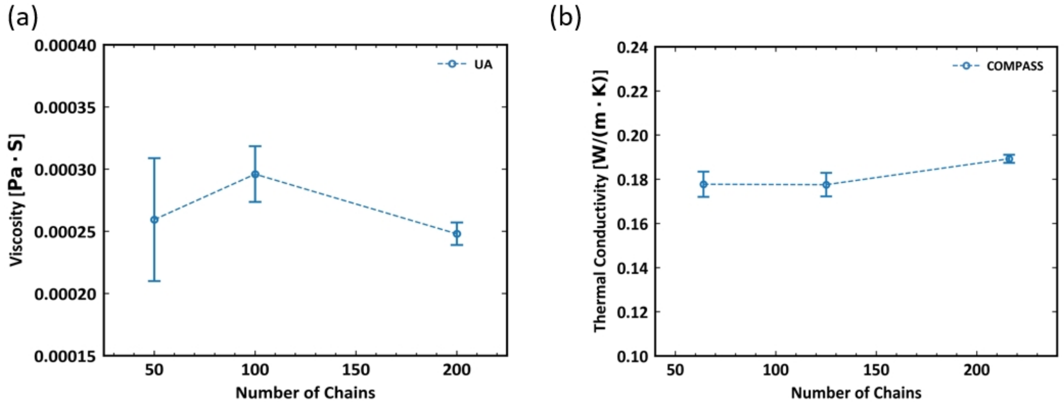

Figure S3: (a) Viscosity using the UA force field for a system with 11 monomers as a function of the number of chains at 450 K. (b) Thermal conductivity using the COMPASS force field for a system with 50 monomers as a function of system volume at 300 K.

Comparison between computational speed of various force fields

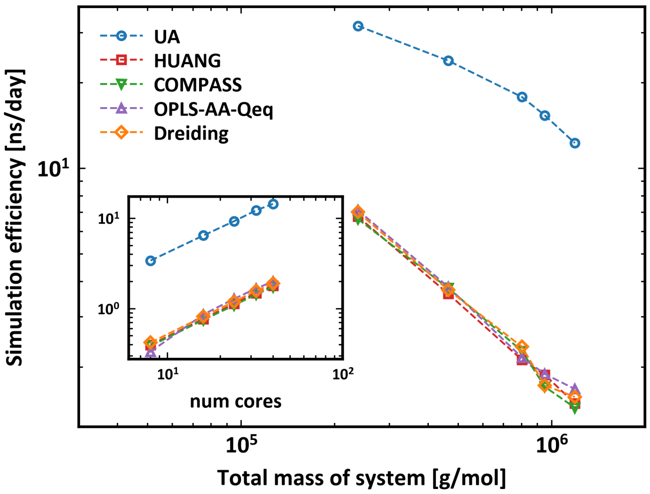

Figure S4: Simulation efficiency in unit of nanosecond per day as a function of total mass of the system in double-logarithmic scaling with a constant number of cores 32; The inset: Timesteps per second as a function of number of cores with a fixed system size comprised of 320 chains, each with 50 monomer.

Details on Experimental Measurements

Density Test

The density of silicone oil is measured using the vibrating tube method, with the equipment model Anton Paar DMA 5000M. Density is an important physical quantity. The definition of liquid density is as follows:

$$\rho = \frac{m}{V}$$

where m is the mass of the liquid, and V is the volume of the liquid. Under the condition that other factors remain unchanged, density does not vary with changes in mass and volume, reflecting the inherent properties of the liquid.

A U-type vibrating tube density meter determines the density of a fluid by measuring the oscillation frequency of a hollow vibrating tube filled with the sample. The resonance frequency, τ, of the vibrating tube depends on the combined mass of the tube and the fluid. The relationship between the density of the fluid, $\rho(T, P)$, at certain temperature T and pressure P, and the measured frequency is given by:

$$\rho\left(T, P\right) = A\left(T, P\right)\tau^2 - B\left(T, P\right) \quad (1)$$

where $A(T, P)$ and $B(T, P)$ are calibration constants determined experimentally using reference fluids of known density. These two calibration constants are defined as follows:

$$A = \frac{K\left(T, P\right)}{4\pi^2 V\left(T, P\right)} \quad (2)$$

$$B = \frac{M_0}{V\left(T, P\right))} \quad (3)$$

where K is the stiffness of the tube, V is the inner volume of the tube, and M_0 is the mass of the evacuated tube. The procedure involves first calibrating the instrument with standard liquids (e.g., distilled water and a calibration oil) to determine $A(T, P)$ and $B(T, P)$. After

calibration, the meter is filled with the sample fluid, and the oscillation frequency is recorded. The density of the fluid is then calculated using the calibration equation. To ensure accuracy, temperature corrections are often applied since the tube's stiffness and fluid properties can vary with temperature.

Specific heat capacity measurement

The specific heat capacity measurement was conducted using a Differential Scanning Calorimeter (DSC), model DSC250 from TA Instruments, USA. The heating rate was set at 10°C/min. Specific heat capacity refers to the amount of heat absorbed or released by a unit mass of material as its temperature changes. During the measurement process, the sample and the reference material are maintained in a state of thermal equilibrium. The instrument continuously monitors the temperature difference between the sample and the reference, and it automatically adjusts the heating power to ensure that their temperature changes synchronously, thereby maintaining thermal equilibrium. This ensures accurate measurement of the heat absorbed or released by the sample at different temperatures.
The specific heat capacity is calculated using the following formula:

$$c = \frac{1}{m}\frac{dQ}{dt}\frac{dt}{dT} \tag{4}$$

Where: c is the specific heat capacity in kJ/(kg·K), Q is the heat in kJ, m is the mass in kg, t is the time in seconds, T is the temperature in Kelvin.

Thermal Conductivity Test

The thermal conductivity of silicone oil is measured using the transient hot wire method, with the DRL-III thermal conductivity tester of the equipment model from Xiangtan Xiangyi Instrument Co., Ltd.

The transient hot-wire method involves using a thin wire as a heat source to apply heat

to the material for a specific duration. As the wire heats up, it raises the temperature of the surrounding material. By monitoring the temperature change of the wire, the thermal conductivity of the material can be calculated. The basic working equation is as follows:

$$\lambda = \frac{q}{4\pi \frac{d\Delta T_{\mathrm{id}}}{d\ln t}} \tag{5}$$

where λ is the thermal conductivity of the liquid, with units W/(m·K), q is the heating power per unit length of the line heat source, with units W/m, ΔT_{id} is the surface temperature rise of the line heat source, with units K, $\ln t$ is the natural logarithm of time, with t in seconds, d is the differential operator.

Dynamic Viscosity Test

The dynamic viscosity of silicone oil is measured using the rotational method, with the equipment model Haake Rheometer Mars60, at a shear rate of 40 1/s.

References

(1) Hanwell, M. D.; Curtis, D. E.; Lonie, D. C.; Vandermeersch, T.; Zurek, E.; Hutchison, G. R. Avogadro: An Advanced Semantic Chemical Editor, Visualization, and Analysis Platform. *Journal of Cheminformatics* **2012**, *4*, 17.

(2) Frischknecht, A. L.; Curro, J. G. Improved United Atom Force Field for Poly(Dimethylsiloxane). *Macromolecules* **2003**, *36*, 2122–2129.

(3) Huang, H.; Cao, F.; Wu, L.; Sun, H. All-Atom and Coarse-Grained Force Fields for Polydimethylsiloxane. *Molecular Simulation* **2017**, *43*, 1513–1522.

(4) Rackers, J. A.; Wang, Z.; Lu, C.; Laury, M. L.; Lagardère, L.; Schnieders, M. J.; Piquemal, J.-P.; Ren, P.; Ponder, J. W. Tinker 8: Software Tools for Molecular Design. *Journal of Chemical Theory and Computation* **2018**, *14*, 5273–5289.

(5) Rappe, A. K.; Goddard, W. A. I. Charge Equilibration for Molecular Dynamics Simulations. *The Journal of Physical Chemistry* **1991**, *95*, 3358–3363.

(6) Frisch, M. J.; Trucks, G. W.; Schlegel, H. B.; Scuseria, G. E.; Robb, M. A.; Cheeseman, J. R.; Scalmani, G.; Barone, V.; Mennucci, B.; Petersson, G. A.; et al. Gaussian 09, Revision D.01. Gaussian, Inc.: Wallingford, CT, 2013.
